# Inter-Strain Differences in LINE-1 DNA Methylation in the Mouse Hematopoietic System in Response to Exposure to Ionizing Radiation

**DOI:** 10.3390/ijms18071430

**Published:** 2017-07-04

**Authors:** Isabelle R. Miousse, Jianhui Chang, Lijian Shao, Rupak Pathak, Étienne Nzabarushimana, Kristy R. Kutanzi, Reid D. Landes, Alan J. Tackett, Martin Hauer-Jensen, Daohong Zhou, Igor Koturbash

**Affiliations:** 1 Department of Environmental and Occupational Health, University of Arkansas for Medical Sciences, Little Rock, AR 72205, USA; iracinemiousse@uams.edu (I.R.M.); kristy.kutanzi@gmail.com (K.R.K.); 2 Department of Pharmaceutical Sciences, Division of Radiation Health, University of Arkansas for Medical Sciences, Little Rock, AR 72205, USA; jchang@uams.edu (J.C.); lshao@uams.edu (L.S.); rpathak@uams.edu (R.P.); mhjensen@uams.edu (M.H.-J.); dzhou@uams.edu (D.Z.); 3 Department of Bioinformatics, School of Informatics and Computing, Indiana University, Bloomington, IN 47408, USA; enzabaru@indiana.edu; 4 Department of Biostatistics, University of Arkansas for Medical Sciences, Little Rock, AR 72205, USA; rdlandes@uams.edu; 5 Department of Biochemistry, University of Arkansas for Medical Sciences, Little Rock, AR 72205, USA; ajtackett@uams.edu

**Keywords:** DNA methylation, hematopoietic cells, ionizing radiation, retrotransposons

## Abstract

Long Interspersed Nuclear Element 1 (LINE-1) retrotransposons are the major repetitive elements in mammalian genomes. LINE-1s are well-accepted as driving forces of evolution and critical regulators of the expression of genetic information. Alterations in LINE-1 DNA methylation may lead to its aberrant activity and are reported in virtually all human cancers and in experimental carcinogenesis. In this study, we investigated the endogenous DNA methylation status of the 5′ untranslated region (UTR) of LINE-1 elements in the bone marrow hematopoietic stem cells (HSCs), hematopoietic progenitor cells (HPCs), and mononuclear cells (MNCs) in radioresistant C57BL/6J and radiosensitive CBA/J mice and in response to ionizing radiation (IR). We demonstrated that basal levels of DNA methylation within the 5′-UTRs of LINE-1 elements did not differ significantly between the two mouse strains and were negatively correlated with the evolutionary age of LINE-1 elements. Meanwhile, the expression of LINE-1 elements was higher in CBA/J mice. At two months after irradiation to 0.1 or 1 Gy of ^137^Cs (dose rate 1.21 Gy/min), significant decreases in LINE-1 DNA methylation in HSCs were observed in prone to radiation-induced carcinogenesis CBA/J, but not C57BL/6J mice. At the same time, no residual DNA damage, increased ROS, or changes in the cell cycle were detected in HSCs of CBA/J mice. These results suggest that epigenetic alterations may potentially serve as driving forces of radiation-induced carcinogenesis; however, future studies are needed to demonstrate the direct link between the LINE-1 DNA hypomethylation and radiation carcinogenesis.

## 1. Introduction

DNA methylation is one of the key epigenetic mechanisms that regulate the expression of genetic information [1]. One of the major functions of DNA methylation is the regulation of transcriptional activity of the repetitive elements that comprise up to two-thirds of mammalian genomes. Retrotransposon Long Interspersed Nuclear Element 1 (LINE-1) is the most abundant repetitive element (covering ~20% of mammalian genomes) and is heavily methylated [2]. LINE-1 retrotransposons are driving forces of evolution and critical regulators of the expression of genetic information [3,4,5,6]. The full-length LINE-1 elements consist of a 5′ untranslated region (UTR), a bicistronic open reading frame that encodes two proteins—ORF1 and ORF2, and a 3′-UTR with a poly(A) tail [2,7]. The primate-specific ORF0—an antisense oriented open reading frame that contributes to retrotransposon-mediated diversity—was also recently reported [8]. Evolutionary patterns of LINE-1 are characterized by the continuous replacement of extinct families by more recently evolved families, which predetermines substantial differences between the elements [9]. These differences primarily stem from the 5′-UTRs, and murine LINE-1s were recently classified according to each family’s unique 5′-UTR sequence and evolutionary age [10].

Exposure to ionizing radiation (IR) affects DNA methylation, and LINE-1 in particular [11]. While the effects of exposure to higher doses of IR is usually associated with the loss of LINE-1 DNA methylation [12], and its reactivation and retrotransposition [12,13], studies on exposures to lower-doses of IR, especially at 1 Gy and below, are less conclusive [14,15]. This may be explained by several factors, including the different approaches used to measure LINE-1 DNA methylation and the sensitivity of those approaches to changes caused by IR, the functional units of LINE-1 used for the analysis, and the analysis performed on the whole tissue lysates, while only certain subsets of cells may be particularly sensitive to IR-induced epigenetic alterations [11].

In these regards, the hematopoietic system represents a valuable experimental tool in epigenetic studies on the effects of IR. The hematopoietic system is highly sensitive to IR and is comprised of cells of different lineages that can be isolated to high purity using multiparameter flow cytometry [16]. Some studies indicate that DNA methylation is also critical for the process of hematopoietic stem cell self-renewal, as shown by serial transplantation in experimental mice [17,18]. Recently, epigenetic alterations were proposed to be among the driving mechanisms in hematological malignancies and serve as a causative factor for radiation-induced leukemogenesis [19,20,21,22,23]. It has to be acknowledged, however, that the most recent study has shown that despite the CpG island hypermethylation observed in virtually all cases of acute myeloid leukemia (AML) with wild-type DNA methyltransferase 3A (DNMT3A), this change was not associated with gene silencing and was essentially absent in AMLs with DNMT3A^R882^ mutations [24].

It is generally accepted that hematopoietic stem cells (HSCs) represent the compartment that is the most susceptible to IR. HSCs reside at the top of the hematopoietic system hierarchy, serving as precursors for all differentiated peripheral blood cells [25]. Therefore, radiation damage to HSCs substantially affects subsequent generations of peripheral blood cells, with long-term consequences.

Earlier studies have demonstrated that IR negatively affects the genome and epigenome in the hematopoietic system [22,26,27]. In a recent study from our group, we reported that a pool of hematopoietic stem and progenitor cells (HSPCs) was more susceptible to epigenetic alterations than mononuclear cells (MNCs) [22]. Furthermore, in the same study, we showed that exposures to leukemogenic doses of IR, while not inducing any detectable long-term DNA damage to HSPCs, are capable of causing long-term epigenetic alterations. The latter included aberrant DNA methylation and expression of LINE-1 and DNA methylation machinery.

These studies experienced a number of limitations, since DNA methylation was measured only within the ORF-1 of LINE-1 elements, while the majority of potential sites for DNA methylation are located within the 5′-UTR. Furthermore, these analyses were performed from the total pool of HSPCs, while substantial differences between the epigenetic profiles of HSCs and hematopoietic progenitor cells (HPCs) have been documented [17,28]. Therefore, in this study, we aimed to investigate the DNA methylation status within the 5′-UTR of LINE-1 elements in HSCs, HPCs, and MNCs of relatively radioresistant C57BL/6J and radiosensitive CBA/J mice in response to IR.

## 2. Results

### 2.1. Comparative Analysis of Hematopoietic Profiles in Non-Irradiated Mice

**Figure 1:**
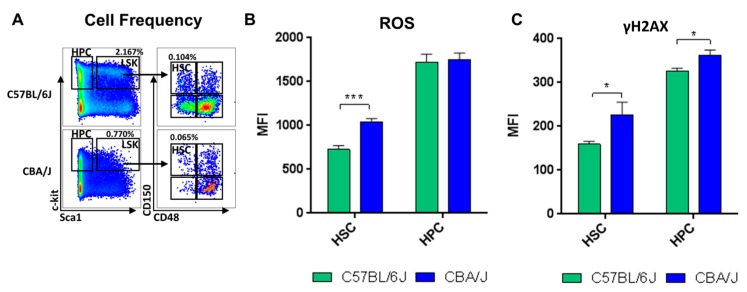
Comparative analysis of hematopoietic profiles of C57BL/6J and CBA/J mice. (**A**) Cell distribution and frequency of hematopoietic stem cells (HSCs) in hematopoietic stem and progenitor cells (HSPCs) (**B**) intracellular levels of endogenous reactive oxygen species (ROS) as measured by fluorescence intensity of 2′7′-dichlorofluorescein (**C**) DNA strand breaks (as measured by fluorescence intensity of γH2AX). Asterisks (*) denote significant (*p* < 0.05) and (***)—(*p* < 0.001) difference from control. LSK: fraction of lineage negative, Sca1 positive, c-kit negative (LSK-) cells, from which HSCs were sorted (*n* = 5/group).

After cells were sorted by FACS based on their expression of membrane proteins, strain differences were noted between C57BL/6J and CBA/J mice. In sham-irradiated animals, consistently lower numbers of total bone marrow cells were collected from CBA/J animals than from C57BL/6J. Additionally, bone marrows from sham-irradiated CBA/J mice yielded fewer HSPCs (bone marrow cells depleted of differentiated cells) than bone marrows from C57BL/6J mice. Furthermore, there was a lower percentage of HSCs in CBA/J (0.06% versus 0.1% in C57BL/6J; *p* < 0.01) (Figure 1A).

The percentage of HSCs in G0 phase was not significantly different between the two strains (*p* = 0.08), while the percentage of HPCs in G0 was significantly higher in CBA/J mice (*p* = 0.006) (Figure S1). The basal levels of ROS, as expressed by DCF-DA fluorescence, were higher in HSCs of CBA/J mice compared to HSCs of C57BL/6J mice (*p* < 0.001) (Figure 1B). The endogenous levels of γH2A.X were also considerably higher in CBA/J mice compared to C57BL/6J in both HSCs and HPCs (*p* < 0.05) (Figure 1C).

### 2.2. Comparative Analysis of LINE-1 Methylation in the Hematopoietic System in Non-Irradiated Mice

Recent advances in computational biology have led to the classification of LINE-1 elements based on their evolutionary age and 5′-UTR sequences [10]. However, analyses of repetitive sequences on the global scale are challenging, given their copy-numbers and polymorphic features. Therefore, we have recently developed a real-time PCR assay that allows for simultaneous analysis of DNA methylation of all 29 currently known LINE-1 elements. Using this approach, we have recently reported changes in the DNA methylation of selective LINE-1 elements after exposure to dense IR [29,30]. Considering the high abundance of LINE-1 elements in the genome, we adapted this approach for this study to perform LINE-1 DNA methylation analysis with a starting material of only 500 cells (described in detail in the Materials and Methods section).

First, we analyzed the endogenous levels of DNA methylation in LINE-1 elements in HSCs and MNCs of C57BL/6J mice. We identified a negative correlation between the basal methylation status of LINE-1 elements and their evolutionary age (slope significantly non-zero, *p* < 0.0001) (Figure S2). LINE-1 elements that were ~10 Myr of age or more were methylated at about one-third of the level found in elements that were less than 1 Myr in age. Importantly, this trend was observed in all evaluated cell types, suggesting it is universal across all the cell types within the hematopoietic system.

**Figure 2:**
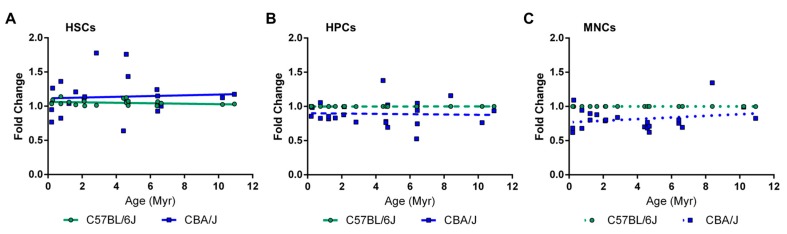
Comparative analysis of LINE-1 5′-UTR DNA methylation in C57BL/6J and CBA/J mice. LINE-1 DNA methylation was measured in (**A**) hematopoietic stem cells (**B**) hematopoietic progenitor cells, and (**C**) mononuclear cells using the adapted method for 500-cell methylation-sensitive real time-PCR (RT-PCR). Data are presented as mean ±SEM (*n* = 5) and show the linear regression between DNA methylation and evolutionary age of the element (Myr, million years). Values from C57BL6/J animals were arbitrarily given a value of 1 for the purpose of comparison (*n* = 5/group).

We then compared the levels of DNA methylation in C57BL/6J and CBA/J mice in HSCs, HPCs, and MNCs (Figure 2, panels A, B, and C, respectively). We arbitrarily set the methylation levels to 1 in all elements from C57BL/6J to highlight strain differences. We did not identify any substantial differences between CBA/J and C57BL/6J.

### 2.3. Comparative Analysis of LINE-1 Expression and DNA Methylation Machinery Expression in the Hematopoietic System in Non-Irradiated Mice

**Figure 3:**
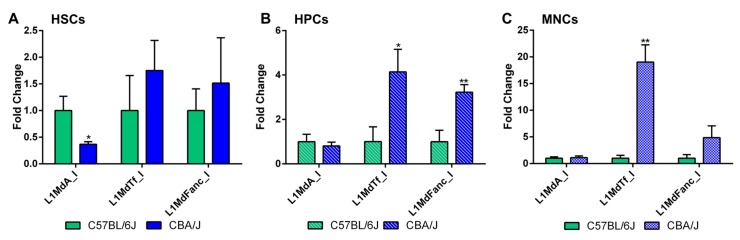
Comparative analysis of expression of three evolutionary distinct LINE-1 elements in the hematopoietic system of C57BL/6J and CBA/J mice: L1MdA_I (0.21 Million years, Myr); L1MdTf_I (0.25 Myr), an L1MdFanc_I (6.80 Myr). The differential expression of LINE-1 elements was determined by quantitative RT-PCR for HSCs (**A**), HPCs (**B**), and MNCs (**C**). Data are presented as mean ± SEM (*n* = 5). Asterisks (*) denote significant (*p* < 0.05), and (**)—(*p* < 0.01) difference from the control. Values from C57BL6/J animals were arbitrarily given a value of 1 for the purpose of comparison (*n* = 5/group).

Differences were noted between the C57BL/6J and CBA/J mice in the expression of LINE-1 elements. Specifically, the expression of the youngest LINE-1 element in the mouse genome, L1MdA_I, was 2.2-fold higher (*p* < 0.05) in HSCs of C57BL/6J mice compared to CBA/J mice (Figure 3A). At the same time, L1MdTf_I and the ancient L1MdFanc_I were more highly expressed in HPCs and MNCs of CBA/J mice (Figure 3B,C) compared to C57BL/6J mice: 4.1-fold (*p* < 0.05) for L1MdTf_I and 3.6-fold (*p* < 0.05) for L1MdFanc_I in HPCs; 18.3-fold (*p* < 0.01) for L1MdTf_I and trending towards an increase for L1MdFanc_I (4.6-fold, *p* = 0.09) in MNCs. No significant differences were detected in the expression of *Dnmt1*, *Dnmt3a*, and *Uhrf1*—enzymes needed for the maintenance of normal patterns of DNA methylation—between the two strains of mice (data not shown).

### 2.4. Long-Term Effects of Exposure to Ionizing Radiation on the Mouse Hematopoietic System

**Figure 4:**
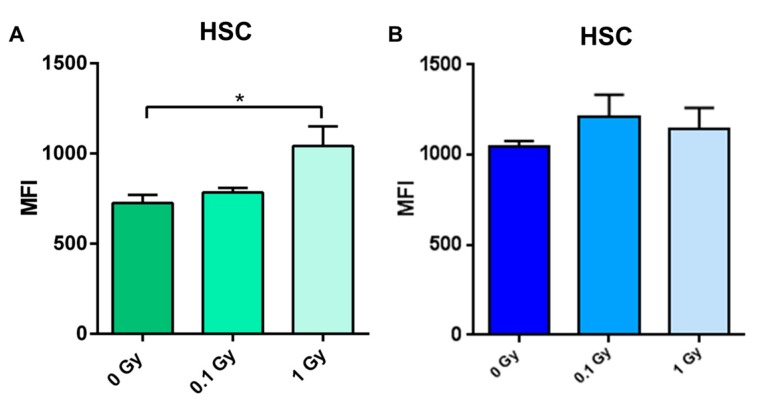
Comparative analysis of the levels of reactive oxygen species in response to low-dose radiation. Mean fluorescence intensity (MFI) for 2’,7’ –dichlorofluorescin diacetate (DCF-DA) in HSCs of C57BL/6J (**A**) and CBA/J (**B**) mice. Asterisks (*) denote significant (*p* < 0.05) difference from control (*n* = 5/group).

To investigate the long-term effects and possible inter-strain differences in response to IR, bone marrows were harvested from C57BL/6J and CBA/J mice two months after total body irradiation (TBI) with either 0.1 or 1 Gy of ^137^Cs. Significant increases were observed in ROS in HSCs of C57BL/6J, but not CBA/J mice, after exposure to 1 Gy (1.5-fold increase, *p* < 0.05) (Figure 4). No significant radiation-induced changes in DNA damage or cell cycle were observed in either strain (data not shown).

### 2.5. Long-Term Effects of Exposure to Ionizing Radiation on LINE-1 DNA Methylation in the Hematopoietic System

**Figure 5:**
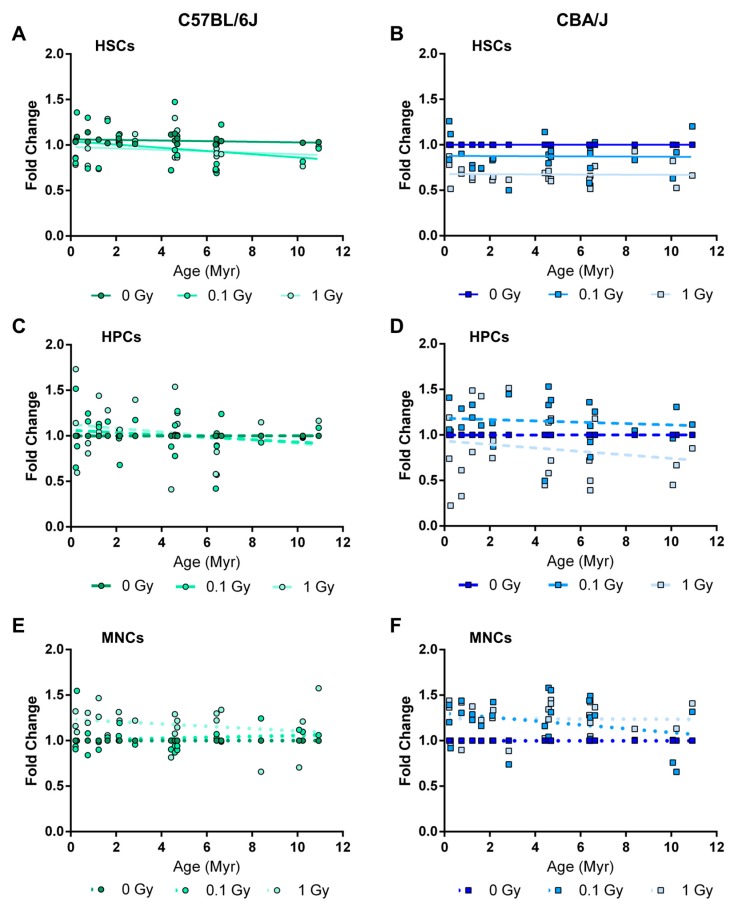
Comparative analysis of LINE-1 DNA methylation in the hematopoietic system of C57BL/6J and CBA/J mice in response to low-dose radiation. LINE-1 DNA methylation was analyzed in (**A**,**B**) hematopoietic stem cells, (**C**,**D**) hematopoietic progenitor cells, and (**E**,**F**) mononuclear cells in C57BL/6J (**A**,**C**,**E**) and CBA/J (**B**,**D**,**F**). Sham-irradiated samples (0 Gy) were arbitrarily given a value of 1 for the purpose of comparison (*n* = 5/group). Myr: million years.

Next, we analyzed the extent of DNA methylation in LINE-1 elements two months after TBI. Exposure to both 0.1 and 1 Gy of ^137^Cs led to a loss of LINE-1 DNA methylation in both mouse strains; however, the hypomethylated phenotype was more evident in HSCs of CBA/J mice (Figure 5B). While the loss of DNA methylation in LINE-1 elements of C57BL/6J mice was comparatively small (up to 12%), LINE-1 DNA hypomethylation in CBA/J mice was much more substantial (up to ~60% compared with basal levels) (Figure 5B). Furthermore, a significant difference in DNA methylation (*p* < 0.0001) was observed between 0 Gy and 1 Gy of TBI in HSCs from CBA/J animals. The loss of DNA methylation in HSCs was not promoter type-dependent or evolutionary age-dependent; however the methylation status of the L1MdA_I—the youngest (0.21 Myr) and the most retrotranspositionally active LINE-1 element—was two-fold lower (*p* < 0.001) after exposure to 1 Gy, compared to sham irradiated mice.

In HPCs of C57BL/6J mice, TBI did not cause any detectable changes in DNA methylation of LINE-1 elements (Figure 5C). In CBA/J mice, exposure to 0.1 Gy resulted in apparent DNA hypermethylation, while exposure to 1 Gy led to DNA hypomethylation of LINE-1 elements. However, intra- and inter-individual variability was larger than in HSCs or MNCs.

In MNCs, TBI led to an increase in the levels of LINE-1 DNA methylation. In CBA/J mice, LINE-1 DNA hypermethylation was noticeable after both 0.1 and 1 Gy of TBI. A significant difference in LINE-1 DNA methylation between 0 Gy and 1 Gy of TBI in MNCs from CBA/J mice was observed (*p* < 0.0001) (Figure 5B). The effect was not significant in C57BL/6J. None of the slopes were found to be significantly different from zero, unsupportive of a role of the age of the element in response to TBI.

### 2.6. Long-Term Effects of Exposure to Ionizing Radiation on LINE-1 and DNA Methylation Machinery Expression in the Hematopoietic System

**Figure 6:**
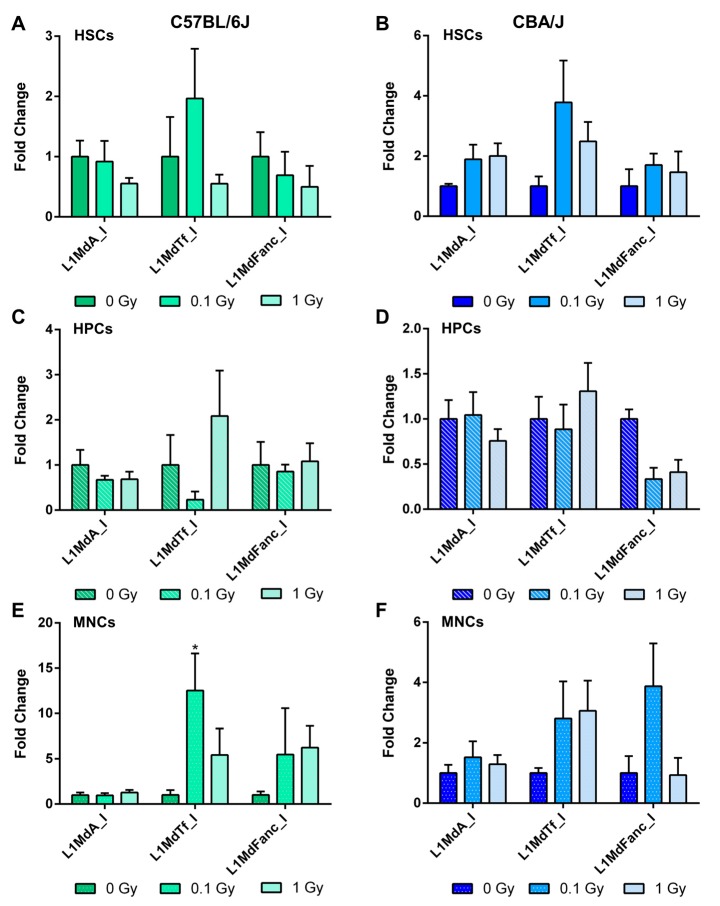
Comparative analysis of expression of three evolutionary distinct LINE-1 elements in the hematopoietic system of C57BL/6J and CBA/J mice in response to low-dose radiation. The differential expression of LINE-1 elements was determined by quantitative RT-PCR in (**A**,**B**) hematopoietic stem cells, (**C**,**D**) hematopoietic progenitor cells, and (**E**,**F**) mononuclear cells in C57BL/6J (**A**,**C**,**E**) and CBA/J (**B**,**D**,**F**). Data are presented as mean ±SEM (*n* = 5). Asterisks (*) denote significant (*p* < 0.05) difference from control. Sham-irradiated samples (0 Gy) were arbitrarily given a value of 1 for the purpose of comparison (*n* = 5/group).

The effects of exposure to IR on the expression of LINE-1 elements were less substantial than on LINE-1 DNA methylation. We evaluated the transcript levels for three LINE-1 elements: L1MdA_I, L1MdTf_I, and L1MdFanc_I. No significant changes were observed for either HSCs or HPCs (Figure 6A–D). A significant 12.4-fold increase in the expression of L1MdTf_I (*p* < 0.05) was observed in MNCs of C57BL6/J mice after exposure to 0.1 Gy of ^137^Cs (Figure 6E). In the three analyzed LINE-1 elements, there were no correlations between the levels of LINE-1 DNA methylation and their expression. No significant changes in the expression of DNA methylation machinery were observed (Figure S3).

## 3. Discussion

In this study, we investigated the differences in the hematopoietic profiles of two mouse strains—the relatively radioresistant C57BL/6J and relatively radiosensitive CBA/J—under normal conditions and in response to IR. In sham-irradiated animals, consistently lower total bone marrow cells were collected, and a smaller percentage of the sorted HSPCs displayed stem-specific cell surface markers from CBA/J animals than those collected from C57BL/6J. These rarer, CBA/J-derived HSCs, also displayed higher basal levels of markers for ROS and γH2AX, even without IR exposure. In fact, the basal level of the ROS marker DCF-DA in HSCs from the CBA/J strain were comparable to the levels of ROS found in C57BL/6J mice exposed to 1 Gy of radiation. At the same time, no significant long-term increases in ROS levels were observed in CBA/J mice in response to either dose of IR. We also assessed γH2AX as a measure of DNA damage. We did not observe any residual DNA damage two months after exposure to IR, consistent with other studies on DNA double-strand break repair kinetics [31,32].

DNA methylation is a key epigenetic mechanism regulating the expression of genetic information. It is also crucial for the normal process of hematopoiesis [16,17,18,19]. Furthermore, studies indicate that epigenetic alterations may play a driving role in radiation-induced experimental carcinogenesis [21,22,33]. In addition, mutations in the DNA methyltransferases DNMT1 and DNMT3A resulted in alterations in the hematopoietic system and may lead to malignant transformation, emphasizing the importance of DNA methylation in hematopoietic malignancies [34,35].

The epigenetic effects of exposure to doses of IR above 1 Gy have been investigated relatively well and are usually characterized by a loss of global and LINE-1-associated DNA methylation [12,21,26]. At the same time, several studies reporting the effects of exposure at doses below 1 Gy are less conclusive. Lacks of changes, as well as both DNA hypo- and hypermethylation, have been reported [14,15,22,30,33,36,37,38,39,40]. Therefore, in this study, we aimed to investigate the DNA methylation of LINE-1 elements in their 5′-UTR in FAC-sorted HSCs, HPCs, and MNCs of two mouse strains with different sensitivity to IR.

Because of the limited number of HSCs that can be obtained from the mouse bone marrow, a methylation-sensitive quantitative real-time PCR (MS qRT-PCR) analysis of LINE-1 with 500 cells as starting material was developed. There are several thousand copies of each family of LINE-1 elements per cell, providing a strong initial signal. Although other techniques exist, such as tagmentation-based whole-genome bisulfite sequencing, they usually require ~10,000 FAC-sorted cells, as well as time- and resource-consuming bioinformatic analysis [41]. Our approach allows for evaluation of repetitive elements-associated DNA methylation from individual mice, substantially decreasing the number of mice needed, as well as the overall time and cost of the experiment.

Consistent with previous findings in human embryonic cells [42] and mouse lungs [29], the degree of LINE-1 element DNA methylation within the 5′-UTR strongly correlated with their evolutionary age in C57BL/6J mice. The 5′-UTRs of the older LINE-1 elements were significantly less methylated when compared to evolutionary younger LINE-1 elements. Importantly, these trends were observed in cells of different lineages that, together with the results of previous studies [22,29], suggested the universal nature of this effect. The differences in DNA methylation observed here for the same LINE-1 elements between HSCs and MNCs were minimal, and suggested that the decrease in global DNA methylation observed during differentiation [28] may stem from regions outside of repetitive elements. Similarly, subtle differences were observed between LINE-1 elements in cells of the same lineage between the two mouse strains—C57BL/6J and CBA/J.

The observed lower DNA methylation in older LINE-1 elements may partially be due to the higher number of acquired mutations in evolutionary older elements compared to evolutionary younger elements. It is known that 5-methylcytosine is prone to spontaneous deamination, resulting in the loss of the methyl group due to its conversion into thymine. Another possible explanation is that only young LINE-1 elements are capable of retrotransposition, therefore, DNA methylation of 5′-UTR as a key mechanism of regulation is necessary primarily in these elements, as opposed to inactivated and truncated older LINE-1 elements.

Differences were detected in the expression of LINE-1 elements between the two mouse strains. The expression level of the youngest (0.21 Myr) and most transcriptionally active L1MdA_I element in HSCs of C57BL/6J mice was two-fold higher than that of CBA/J mice. The expression of yet another young (0.25 Myr) and potentially retrotranspositionally active L1MdTf_I element was also higher in HPCs and MNCs from CBA/J mice compared to C57BL/6J mice. These differences were DNA methylation-independent and could be mediated by histone modifications or non-coding RNAs [43]. Differential expression of specific LINE-1 elements may predetermine the cellular plasticity of HSCs and their differentiation/proliferation status. Therefore, future studies will be needed to pinpoint genes located in proximity to LINE-1 elements and their influence on HSC commitment.

Our results also indicate that IR induces lineage-specific changes in LINE-1 DNA methylation of hematopoietic cells. The most pronounced effects were observed in HSCs, reported as the most sensitive to IR [44]. In this regard, the results of our study highlight several important facts. First, in HSCs of CBA/J mice, epigenetic alterations that are characterized by increased susceptibility to radiation-induced leukemia [45] were detected long after exposure to IR (2 months), suggesting the persistent nature of the effect. Persistence is a prerequisite for epigenetic alterations to be considered as driving forces of radiation-induced carcinogenesis [46,47]. Second, the loss of LINE-1 DNA methylation observed in HSCs two months after irradiation was independent of DNA damage, induction of ROS, or cellular senescence. Similar findings were observed in HSPCs 22 weeks after exposure to a leukemogenic dose of ^56^Fe [22]. This suggests that epigenetic alterations in radiation-induced carcinogenesis may arise without persistent genetic damage, particularly at low doses. Third, these changes were observed in a strain that is recognized for its sensitivity to radiation-induced leukemogenesis (CBA/J), but not in a reference strain (C57BL/6J).

Loss of epigenetic control over LINE-1 is linked to its aberrant transcriptional activity, insertional mutagenesis, development of genomic instability, and carcinogenesis [48]. Indeed, alterations in LINE-1 DNA methylation are reported in most clinical and experimental cancers [2]. The role of LINE-1 in hematopoietic malignancies has also been recognized [49].

Numerous studies have reported radiation-induced alterations in DNA methylation and expression of LINE-1 [29,30,37,38]. In this study, we identified few significant changes in the expression of the three tested LINE-1 elements two months after exposure to IR. However, because of the large number of copies and long evolutionary history of retrotransposons, numerous genes acquired LINE-1 insertions within their coding sequence and regulatory units. Therefore, alterations in DNA methylation within the LINE-1 elements may have affected the expression of their host genes [50,51]. In this regard, more studies are needed to investigate the relationship between the methylation status of LINE-1 insertions and the expression status of host genes.

This study has several limitations. The starting material of 500 cells provided enough material to analyze the DNA methylation of most of the reported LINE-1 families. However, their expression was relatively low and we were only able to investigate a subset for transcript levels. We selected three LINE-1 elements that belong to structurally different promoter types: A (L1MdA_I), Tf (L1MdTf_I), and Fanc (L1MdFanc_I), and are strikingly different by age—from the 0.21 Myr old L1MdA_I to 6.8 Myr old L1MdFanc_I. We cannot exclude that some other LINE-1 elements not included in the analysis could be more responsive to IR; therefore, further studies will be needed to investigate the effects of IR on the transcription status of other LINE-1 elements. Also, both DNA methylation and the expression of LINE-1 are characterized by a high degree of inter-individual variability that often failed to reach statistical significance. Future studies would benefit from a higher sample size in order to address this issue. Another goal of future studies will be to confirm the causative role of LINE-1 DNA hypomethylation in radiation-induced carcinogenesis. These studies will require development of in vivo models of the hypomethylated LINE-1 phenotype, a functional characterization of genes that evolutionary acquired LINE-1 insertions, and whole-genome sequencing analysis, to identify radiation-induced LINE-1 de novo insertional mutagenesis.

In conclusion, we demonstrated that DNA methylation within the 5′-UTRs of LINE-1 elements significantly differed between the two mouse strains in response to exposure to IR. The most pronounced effects in response to TBI were observed in HSCs, the cells characterized by the highest sensitivity to IR in CBA/J mice prone to radiation-induced leukemogenesis. These effects were characterized by a loss of LINE-1 DNA methylation in HSCs, were persistent in nature, and were maintained without detectable DNA damage. Altogether, these findings suggest that epigenetic alterations are among the driving forces of IR-induced carcinogenesis. Given the considerable plasticity of epigenetic alterations and the potential for DNA methylation modulation by dietary interventions, these findings suggest a potential strategy for the mitigation and possible prevention of negative effects exerted by IR. In this regard, the essential amino acid methionine is of particular interest. It serves as a substrate for synthesis of S-adenosylmethionine (SAM)—the major donor of methyl groups for DNA methylation. Dietary supplementation with methyl donors may aid in sustaining the normal levels of DNA methylation post exposure [52]. For instance, it has been shown that dietary L-methionine supplementation mitigated the radiation-induced loss of DNA methylation in the mouse liver [53]. Future studies will be needed to confirm the beneficial effects of dietary modulation of normal tissue response to IR.

## 4. Materials and Methods

### 4.1. Animals and Irradiation

Eight-week-old male C57BL/6J and CBA/J mice were purchased from Jackson Laboratory (Bar Harbor, ME, USA). Animals were housed at the University of Arkansas for Medical Sciences (UAMS). After a one-week acclimation period, the mice were randomly divided into three groups (*n* = 5): Sham irradiated or whole-body irradiated to 0.1 or 1 Gy of ^137^Cs. Irradiation was performed with a J. L. Shepherd Mark I (model 25 ^137^Cs irradiator (J. L. Shepherd & Associates, San Fernando, CA, USA)). Un-anesthetized mice were placed in well-ventilated cylindrical Plexiglas chambers (J. L. Shepherd & Associates) divided into six 60° “pie slice” compartments by vertical dividers made of machinable grade T-6061 aluminum with a gold anodized coating. Two chambers were stacked on top of each other and placed on a turntable rotating at 5 rpm in the position furthest from the radiation source, allowing all five mice belonging to the same exposure regimen to be irradiated simultaneously. The average dose rate was 1.21 Gy/min. During the entire experiment, sham-irradiated mice were housed in the same room, but in separate cages from the irradiated mice.

In order to investigate the persistent nature of radiation-induced epigenetic alterations and to minimize the effects of the hematopoietic system recovery, the experiment was terminated two months after irradiation. All animals were killed by cervical dislocation. Hind limbs were collected immediately and stored in 1× PBS supplemented with 2% FBS on ice for further bone marrow cell isolation. All procedures were approved by the Institutional Animal Care and Use Committee at UAMS.

### 4.2. Cell Collection

The bone marrow from each femora and tibia specimen was flushed with 2% FBS-PBS and pooled. The bone marrow suspension was carefully overlaid on a Ficoll solution and centrifuged to exclude multinucleated cells. An aliquot of the total pool of bone marrow mononucleated cells was set aside for Fluorescence Activated Cell Sorting (FACS) and referred to as MNCs in the results. Twenty million of the remaining cells from this pool were incubated with primary rat anti-mouse antibodies against the markers of various differentiated mature hematopoietic cell lineages, including CD3e, B220, Ter119, CD11b, and Gr-1. Differentiated cells were depleted using anti-rat antibody-coated magnetic beads (Dynabeads, Life Technologies, Carlsbad, CA, USA). Each resulting depleted pool containing both stem and progenitor cells (referred to as HSPCs) was counted and one million cells per mouse were used for cell frequency analysis and nucleic acid isolation, and another million cells for ROS, γH2AX, and cell cycle analysis, respectively.

### 4.3. LINE-1 Families Consensus Sequences

LINE-1 families’ consensus sequences were obtained from the Genetic Information Research Institute (GIRI) Database: http://www.girinst.org/ [54,55].

### 4.4. Construction of LINE-1 Phylogenetic Tree

Molecular phylogenetic analysis was conducted in MEGA6 [56] and based on the consensus sequences of the 29 LINE-1 families we analyzed. First, the consensus sequences were aligned using MUSCLE 3.8.31 software with default settings [57]. Next, the evolutionary history of the 29 LINE-1 families was inferred using the Maximum Likelihood method based on the Hasegawa-Kishino-Yano model [58]. Original trees were obtained by applying Neighbor-Join and BioNJ algorithms to a matrix of pairwise distances estimated using the Maximum Composite Likelihood (MCL) approach, and then selecting the topology with superior log likelihood value. The phylogenetic analysis included 1^st^ + 2^nd^ + 3^rd^ + Noncoding codon positions with a complete deletion option. The final dataset included 4494 positions. The consensus representative phylogenetic tree was obtained using the bootstrap method [59] with 1000 replicates where branches whose partitions are reproduced in less than 50% of bootstrap replicates are collapsed (Figure S4).

### 4.5. Nucleic Acid Isolation and Digestion

RNA and DNA were isolated simultaneously from the 500 FAC-sorted HSCs, HPCs, and MNCs using the AllPrep micro kit (Qiagen, Germantown, MD, USA) according to the manufacturer’s protocol. DNA concentrations and integrity were analyzed by the Nanodrop 2000 (Thermo Scientific, Waltham, MA, USA).

### 4.6. Analysis of DNA Methylation within the 5′-UTR of Selective LINE-1 Elements

First, the 5′-UTRs of twenty nine LINE-1 elements were analyzed using NEBcutter^®^ (http://nc2.neb.com/NEBcutter2/). The five most frequent CpG sites that could be cleaved by the methylation-sensitive restriction enzymes (*Aci*I, *BstU*I, *Hha*I, *Hpa*II, and *Sma*I) were identified. Analysis of DNA methylation was performed as follows: genomic DNA extracted from 500 sorted cells was digested with 1 U of *Sma*I in 1× CutSmart buffer at 25 °C for 2 h. This was followed by a 16 h digestion at 37 °C in the presence of 1 U of the *Hpa*II, *Hha*I, and *Aci*I in 1× CutSmart buffer. The digestion was finalized by adding 0.5 U of *BstU*I in 1× CutSmart buffer for 4 h at 60 °C (New England Biolabs). Digested DNA was then analyzed by qRT-PCR on a ViiA 7 Real-Time PCR System (Applied Biosystems, Foster City, CA, USA) for 29 LINE-1 families. DNA samples not digested with the restriction enzyme mix served as a positive control, while samples lacking the specific primers for DNA amplification or DNA template served as negative controls. The Ct was defined as the fractional cycle number that passes the fixed threshold. The Ct values were converted into the absolute amount of input DNA using the absolute standard curve method, and further normalized towards readings from the respective to each LINE-1 element ORF1 region that lacked CpG sites. We successfully validated assays for 27 out of 29 tested LINE-1 elements. Forward and reverse primers for determination of 5′-UTR LINE-1 DNA methylation are provided in Table S1. Forward and reverse primers for LINE-1 DNA targets and control regions were mixed and aliquoted in a 96-well qPCR plate, so that three samples could be loaded side-by-side on each plate. The total volume of primer solution per well was 3 µL, containing a total of 2.5 µM each of forward and reverse primer (Table S1). The digested DNA was mixed with SYBR Select master mix (Life Technologies) and 7 µL was added to each well. Reactions were performed on a ViiA-7 instrument according to the protocol suggested for the master mix (Applied Biosystems). In order to validate the methylation-sensitive digestion protocol, DNA digested with a cocktail of five methylation-sensitive enzymes was compared to sonicated DNA in HSC and MNCs from C57BL/6J mice. This allowed for the normalization of possible differences in copy numbers, unrelated to DNA methylation.

### 4.7. Gene Expression Analysis

cDNA was synthesized from total RNA sample using the SuperScript ViLO reverse transcriptase kit (Life Technologies) according to the manufacturer’s protocol. Quantitative real-time PCR (qRT-PCR) was performed with Taqman Universal Master Mix (Life Technologies) according to the manufacturer’s protocol. Primers were added at a final concentration of 5 µM (Tables S1 and S2). Expression of *Dnmt1*, *Dnmt3a*, *Uhrf1*, and three LINE-1 elements (L1MdA_I, L1MdTf_I, and L1MdFanc_I) was normalized to the internal control gene *Hprt* and expressed as fold change according to the ΔΔ*C*_t_ method.

### 4.8. Analysis of the Frequencies and Numbers of Different Hematopoietic Cell Populations by Flow Cytometry

Bone marrow HSPCs were stained with biotin-conjugated anti- CD3e, B220, Ter119, CD11b, and Gr-1 antibodies and then with streptavidin-fluorescein isothiocyanate (FITC) and anti-Sca1-PE-Cy7 (clone E13-161.7), c-Kit-APC-Cy7 (clone 2B8), CD150-APC, and CD48-Pacific blue (BD Biosciences, San Diego, CA, USA) fluorescently labeled antibodies. The frequencies of each cell type were analyzed with an Aria II cell sorter. For each sample, approximately 5 × 10^5^ to 1 × 10^6^ HSPCs were acquired and the data were analyzed using BD FACSDiva 6.0 (BD Biosciences) and FlowJo (FlowJo, Ashland, OR, USA) software. Five hundred HSCs, HPCs, and MNCs were sorted for nucleic acid extraction. An additional 500 mononuclear cells were sorted from the pool of cells set aside before depletion.

### 4.9. Analysis of the Levels of Intracellular ROS

After staining with the appropriate cell surface marker antibodies as described above, cells (1 × 10^6^/mL) were suspended in phosphate buffered saline (PBS) (Mediatech Inc., Manassas, VA, USA) supplemented with 5 mM glucose, 1 mM CaCl_2_, 0.5 mM MgSO_4_, and 5 mg/mL bovine serum albumin (BSA), and then incubated with 10 µM 2′,7′-dichlorofluorescin diacetate (DC-FDA) (Life Technologies) for 30 min at 37 °C. The levels of ROS were analyzed by measuring the mean fluorescence intensity (MFI) of 2′,7′-dichlorofluorescein (DCF) with an Aria II cell sorter (Becton-Dickinson, San Jose, CA, USA). For each sample, a minimum of 200,000 cells was acquired, and the data were analyzed using CellQuest software (Becton-Dickinson). R-phycoerythrin (PE) and Allophycocyanin (APC) isotype controls were included, as appropriate.

### 4.10. DNA Damage Analysis

After approximately 4000 sorted cells were first stained with antibodies against specific cell-surface markers as described above, they were fixed and permeabilized using the Fixation/Permeabilization Solution from BD Biosciences (San Diego, CA, USA), followed by 0.2% Triton-X-100 incubation for 10 min. Cells were then stained with Alexa Fluor 488 conjugated anti-phospho-Histone H2AX (Ser139) for 1.5 h at 4 °C and analyzed by flow cytometry. The levels of DNA damage were expressed by the mean fluorescence intensity of γH2AX with an Aria II cell sorter (BD Biosciences).

### 4.11. Statistical Analysis

All animal experiments were performed once. All data are presented as mean ±standard error of mean of five animals per radiation dose. With five animals per comparison group, there was 80% power to detect effect sizes of approximately two standard deviations in a two-tailed *t*-test with a 5% significance level. All assessed parameters were measured within the same batch of animals. Statistically significant differences for each treatment compared to the control were assessed using one-way ANOVAs followed by Dunnett’s or Tukey’s post hoc tests. Statistical analyses were performed using GraphPad Prism 6 (GraphPad Software Inc., LaJolla, CA, USA). For DNA methylation analysis, we performed random coefficient regression to account for multiple observations of LINE-1 DNA methylation (recorded by the LINE-1’s evolutionary age) within a mouse using the MIXED procedure in SAS/STAT software, version 9.3, SAS System for Windows (SAS Institute, Cary, NC, USA).

## Supplementary Materials

Supplementary materials can be found at www.mdpi.com/1422-0067/18/7/1430/s1.

## Abbreviations

FACS	Fluorescence activated cell sorting
HPC	Hematopoietic progenitor cell
HSC	Hematopoietic stem cell
HSPC	Hematopoietic stem and progenitor cell
IR	Ionizing radiation
LINE-1	Long Interspersed Nucleotide Element 1
MNC	Mononuclear cell
Myr	Million years
ORF	Open reading frame
ROS	Reactive oxygen species
SEM	Standard error of mean
TBI	Total body irradiation
UTR	Untranslated region
