# Supplementary material for: Inter-Strain Differences in LINE-1 DNA Methylation in the Mouse Hematopoietic System in Response to Exposure to Ionizing Radiation
Source: Int J Mol Sci. 2017 Jul 4;18(7):1430. doi: 10.3390/ijms18071430 (PMC5535921; doi:10.3390/ijms18071430)
Supplement: Supplementary file 1 [file ijms-18-01430-s001.zip › ijms-205179-supplementary.docx]

**
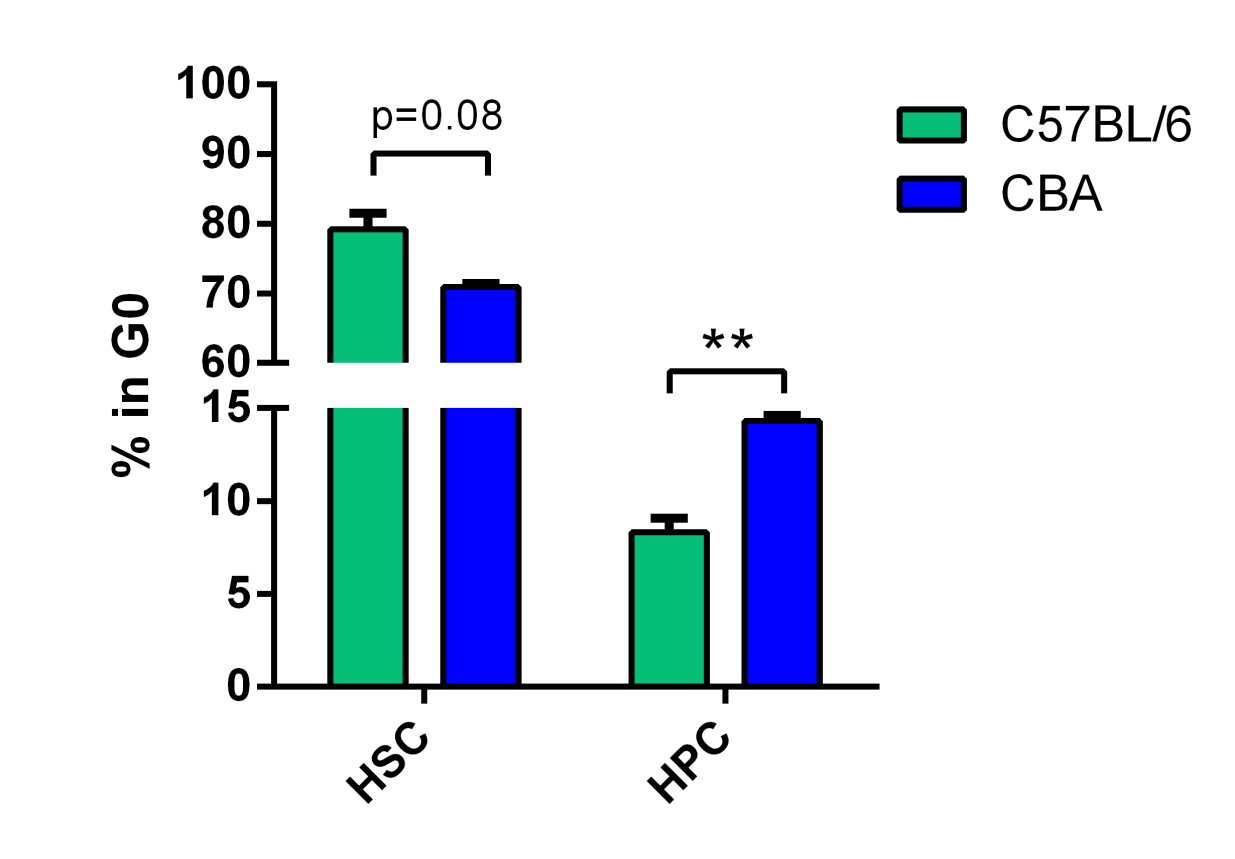
Supplementary Figures**

**Figure. S1.** Percentage of cells in the G0 phase of the cell cycle in hematopoietic stem cells (HSCs) and hematopoietic progenitor cells (HPCs) of C57BL/6J and CBA/J mice. Asterisks (*) denote significant (*p* < 0.05), and (**) - (*p* < 0.01) difference from control.


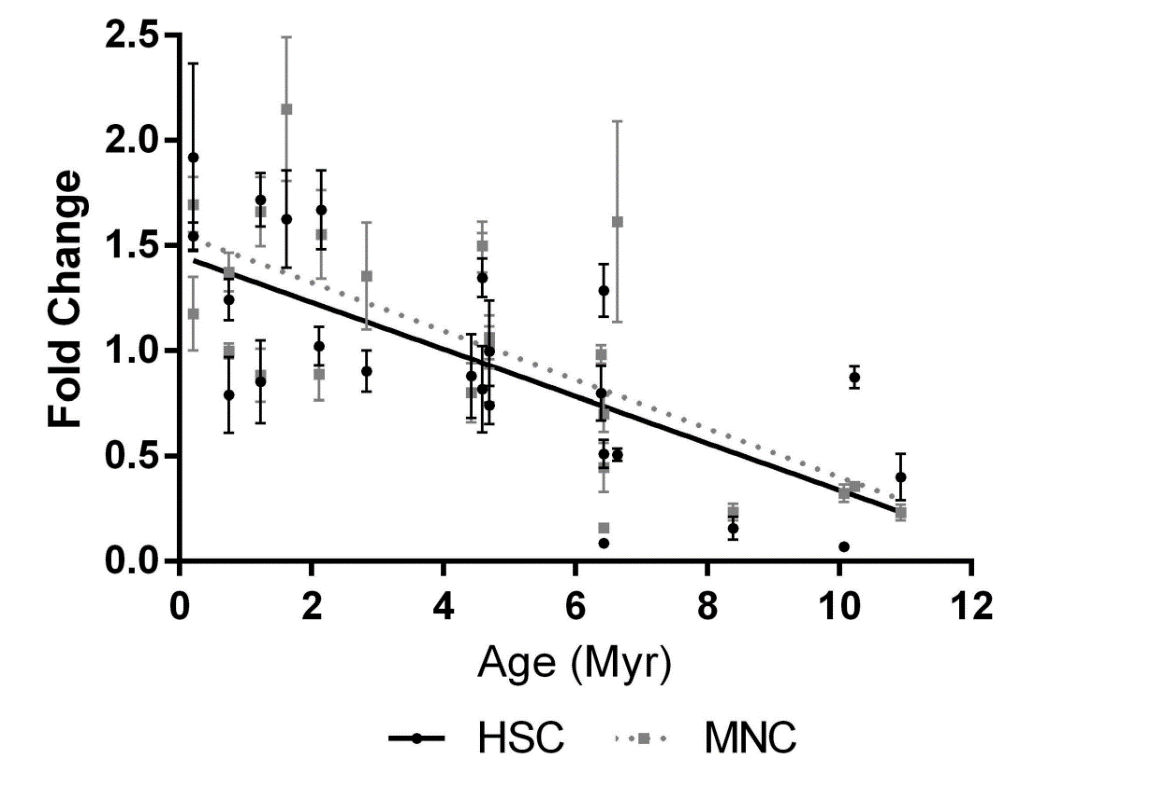


**Figure. S2.** Correlation between the evolutionary age and Long Interspersed Nucleotide Element 1 (LINE-1) DNA methylation in the hematopoietic system of C57BL/6J mice.


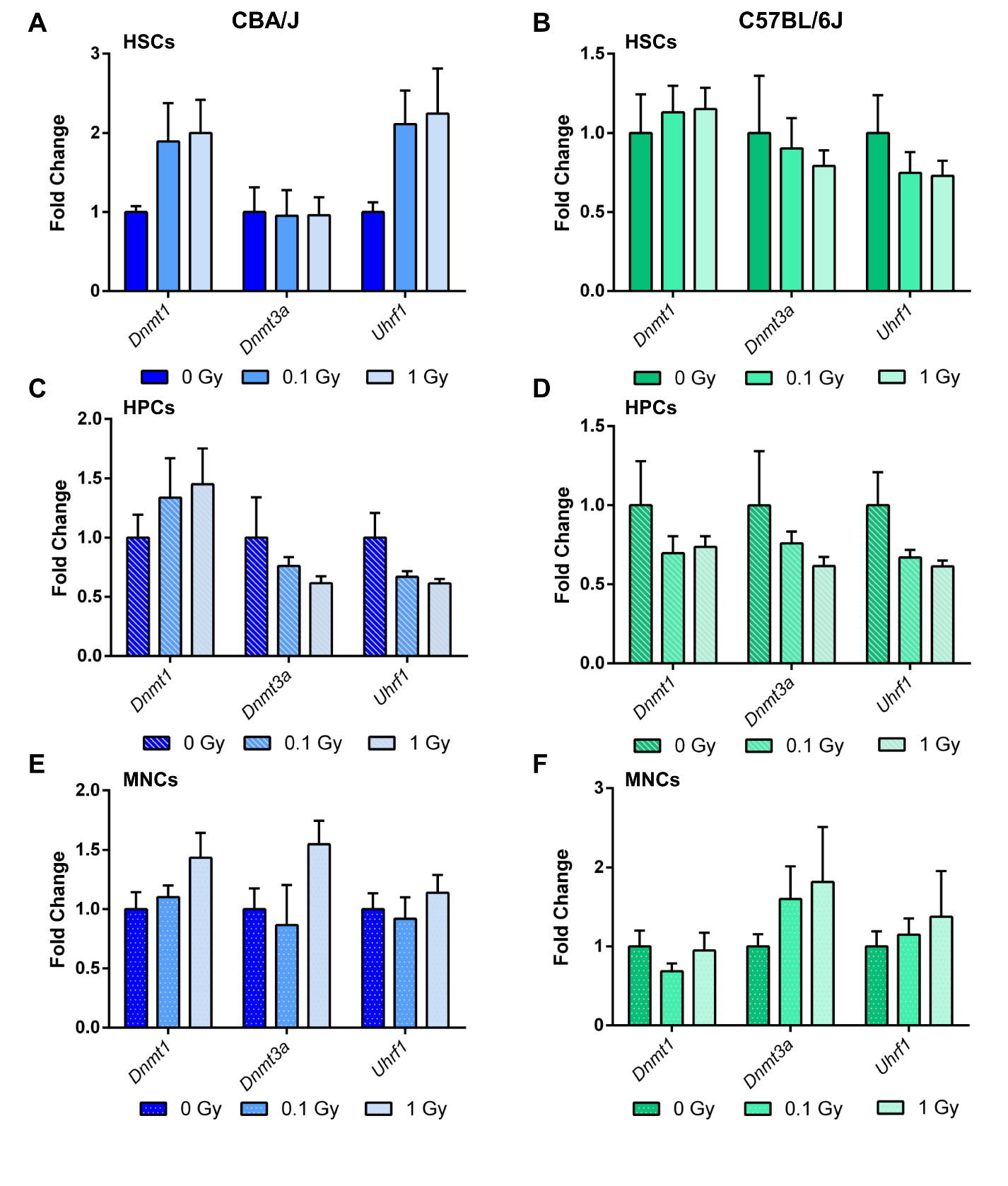


**Figure. S3.** Gene expression for *Dnmt1*, *Dnmt3a*, and *Uhrf1* after exposure to 0, 0.1, and 1 Gy ionizing radiation (IR) in HSCs (panels A and B), HPCs (panels C and D), and mononuclear cells (MNCs) (panels E and F) from CBA/J (panels A, C, and E) and C57BL/6J (panels B, D, and F) mice.


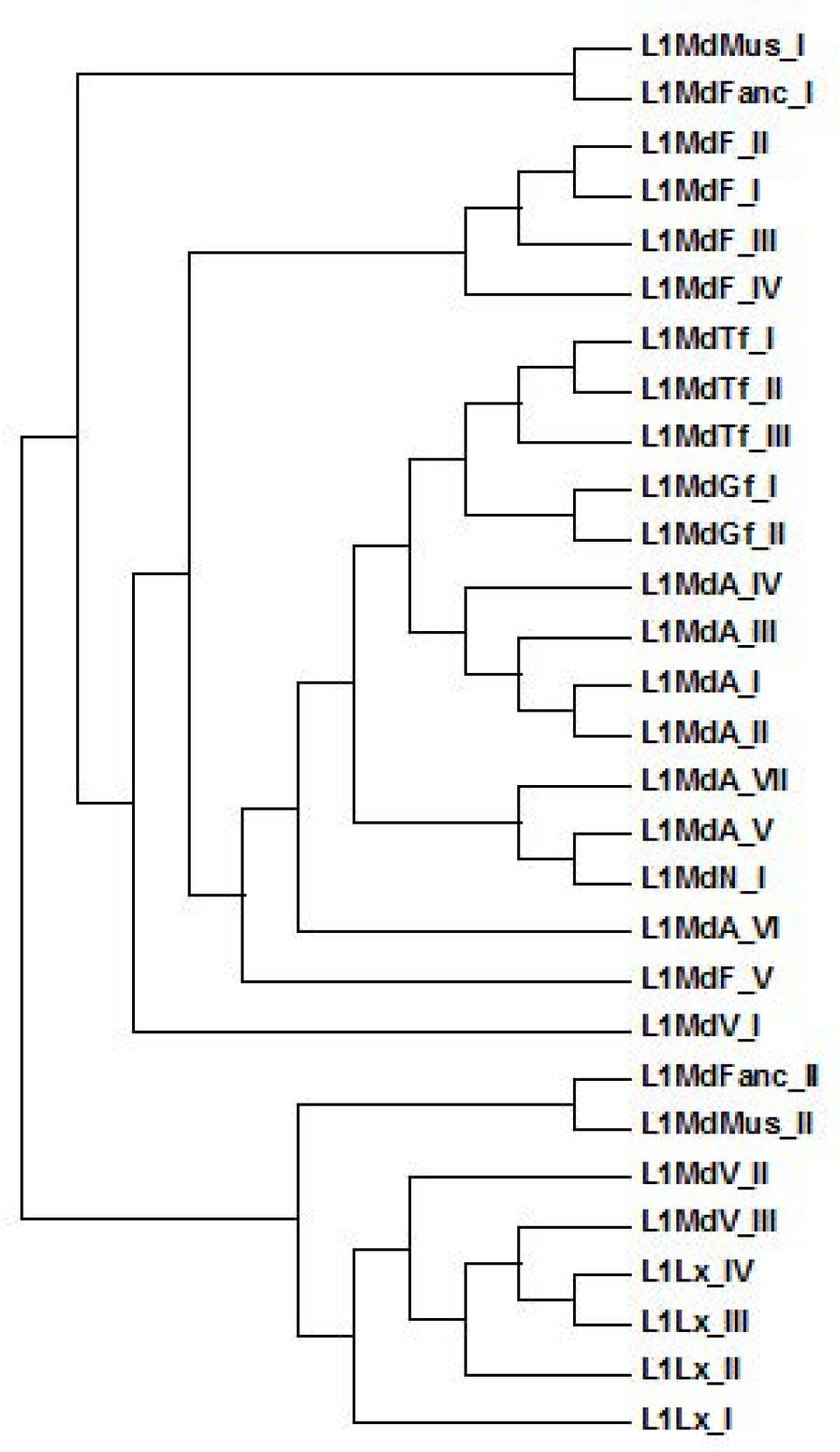


**Figure. S4.** LINE-1 phylogenetic tree. The molecular phylogenetic analysis was conducted in MEGA6 (*29*) and based on the consensus sequences of the 29 LINE1 families analyzed.

**Supplementary Table 1.** Forward and reverse primers for analysis of 5’ untranslated region (UTR) DNA methylation of LINE-1 elements.

| **Family** | **Orientation** | **Sequence** |
| --- | --- | --- |
| L1MdA_I | 5’ | AGTGGATCACAGTGCCTGC |
|  | 3’ | GGGTAGCCTGCTTCCCTATG |
| L1MdA_II | 5’ | ACCTCTGGTGAGTGGAACAC |
|  | 3’ | CAAGACTCTGCTGGCAAGGTA |
| L1MdA_III | 5’ | AAGTCCCTTCCGCTCGACTC |
|  | 3’ | GTACCGCAGTCTCAGGTTCC |
| L1MdA_IV | 5’ | TCTGGTGAGTGGAACACAGC |
|  | 3’ | GAGACTCCGCTGGCAAGG |
| L1MdA_V | 5’ | GGCCCACTGCAGCACC |
|  | 3’ | TTCCTTAATTAATGCAGTCTCAGGT |
| L1MdA_VI | 5’ | AAGTCCCTTCCGGTCCACTC |
|  | 3’ | GCTTCCCTAGTTAATGCAGTCTC |
| L1MdA_VII | 5’ | CCTCTGGTGAGTGGAACACA |
|  | 3’ | CTTAAGATCCCGTGGAGGGTC |
| Tf_I | 5’ | GCAGCACCCTGTGTGGG |
|  | 3’ | GTGCTTCTGCCTGTTCCAGA |
| Tf_II | 5’ | CCTGACAGCTTCTGGAACAGG |
|  | 3’ | TCACCTGTTCAGACTAATTTCCTAA |
| Tf_III | 5’ | CAGCAGCGGTCGCCAT |
|  | 3’ | AGAAGCTGTCCGGTTCTCTG |
| Gf_I | 5’ | CGGGTGACCGTGTGGAAT |
|  | 3’ | GTGCTCTCACCAGGAAGGTGG |
| Gf_II | 5’ | CGGGCGAGAGCCACAG |
|  | 3’ | CGGAGTCCCAGAATCAAGGT |
| L1MdF_I | 5’ | CCTTCCTGGTGAGAGCACAG |
|  | 3’ | GAAACGGCTGGCCTCTGTAT |
| L1MdF_II | 5’ | TCCCGACCAGAGGACAGG |
|  | 3’ | TTTCCTAAGTTCGGCGGAGT |
| L1MdF_III | 5’ | GCTGACAGCTTCTGGGACA |
|  | 3’ | TCTCACCTGTGCAGACTACT |
| L1MdF_IV | 5’ | AAGCTAACAGCTTCTGGGACA |
|  | 3’ | TAGTCCACACTCTCACCTGC |
| L1MdF_V | 5’ | GTTCCAATCCAATCACGCGG |
|  | 3’ | TAGGGGACCTTGGGGGTGTC |
| L1MdFanc_I | 5’ | CAGCTTCTGGGACAGGCAGAA |
|  | 3’ | AGATGTGTTGCCTCTGCAGTCT |
| L1MdFanc_II | 5’ | GTTCCAAAACAACCGGGAGGG |
|  | 3’ | CCGTCCGCCGAGTTCGT |
| L1MdN_I | 5’ | ACTGCATTAGTCAGGGAAGCAA |
|  | 3’ | GCTGTGTTCCACTCACCAGA |
| L1MdV_I | 5’ | CCTGACCAGGGGCACAAG |
|  | 3’ | CTTTGAGGACCGTGGAGCTG |
| L1MdV_II | 5’ | GTCCCCAGAGGACTCTCCAC |
|  | 3’ | GGGGACCGTGGGGCT |
| L1MdMus_I | 5’ | GTGGAACGCAACATCAGCTC |
|  | 3’ | GAGAGTCCTCTGGTGCCCTA |
| L1MdMus_II | 5’ | ACGCAACATCTGTTCCAAAAA |
|  | 3’ | GGTGTGCTAGGGTGCCTG |
| L1MdLx_I | 5’ | CCTCCAGGGAGTGCTCTGA |
|  | 3’ | AGAACAGCCCCACCTCCT |
| L1MdLx_II | 5’ | CCAGAGATAACCAGATGGCGAA |
|  | 3’ | TAAATCCGAATCTTGCTTTTCCG |
| L1MdLx_III | 5’ | TCGCAGGTGTGTAGGCACTC |
|  | 3’ | CCCTCAGGACACAGGAACCG |

| **Target** | **Source** | **Assay ID** |
| --- | --- | --- |
| *Dnmt1* | Life Technologies | Mm01151063_m1 |
| *Dnmt3a* | Life Technologies | Mm00432881 |
| *Dnmt3b* | Life Technologies | Mm01240113_m1 |
| *Uhrf1* | Life Technologies | Mm00477868_mH |
| *Hprt* | Integrated DNA Technologies | Mm.PT.39a.22214828 |

**Supplementary Table 2.** Assays used for gene expression analysis.
